# Population structure, mitochondrial polyphyly and the repeated loss of human biting ability in anopheline mosquitoes from the southwest Pacific

**DOI:** 10.1111/j.1365-294X.2012.05690.x

**Published:** 2012-09

**Authors:** L Ambrose, C Riginos, R D Cooper, K S Leow, W Ong, N W Beebe

**Affiliations:** 1 School of Biological Sciences, University of QueenslandSt Lucia, Brisbane, Qld. 4072, Australia; 2 Australian Army Malaria InstituteBrisbane, Qld. 4051, Australia; 3 CSIRO Ecosystem SciencesBoggo Road, Dutton Park, Qld. 4102, Australia

**Keywords:** Anopheles, introgression, malaria vector, New Guinea, parallel evolution, phylogeography

## Abstract

Australia and New Guinea contain high levels of endemism and biodiversity, yet there have been few evaluations of population-level genetic diversity in fauna occurring throughout the Australo-Papuan region. Using extensive geographical sampling, we examined and compared the phylogenetic relationships, phylogeography and population structure of *Anopheles farauti*, *An. hinesorum* and *An. irenicus* throughout their ranges in the southwest Pacific using mitochondrial (mtDNA COI) and nuclear (ribosomal protein S9 and ribosomal DNA ITS2) loci. Phylogenetic analyses suggest that the ability to utilize humans as hosts has been lost repeatedly, coincident with independent colonizations of the Solomon Islands. As some of the species under investigation transmit malaria in the region, this is a medically important finding. Maximum likelihood and Bayesian phylogenetic analyses of nuclear loci also showed that the three species are monophyletic. However, putative introgression of *An. hinesorum* mtDNA onto a nuclear background of *An. farauti* was evident in populations from Queensland, Torres Strait and southern New Guinea. Haplotype networks and pairwise *F*_ST_ values show that there is significant genetic structure within New Guinea and Australia in both *An. farauti* and *An. hinesorum*, consistent with a long-term history of low gene flow among populations.

## Introduction

### Background

Until recently (about 8000 years ago), Australia and New Guinea were connected by a land bridge (Voris 2000) and the two landmasses are considered part of the same continent known as Sahul (O’Connell & Allen 2004). Now separated, both landmasses support exceptional biodiversity (Allison 2007; Marshall 2007) and there are close affinities between their biotas, reflecting their historical connection (Allison 2007). High levels of local animal endemism are present in parts of New Guinea (Taylor *et al.* 1982; Allison 2007), which may be due to the island’s topographical and geological complexity (Pigram & Davies 1987; Polhemus 2007). This complexity has been the focus of many biogeographical studies on the fauna of New Guinea (Beehler & Swaby 1991; de Boer & Duffels 1996; Polhemus & Polhemus 1998; Heads 2002).

However, despite the importance of the Australo-Papuan region as an evolutionary centre and a biodiversity hotspot (Allison 2007), few studies have investigated population-level patterns of genetic diversity in terrestrial organisms of the area (Rawlings & Donnellan 2003; Murphy *et al.* 2007; Kearns *et al.* 2011; Macqueen *et al.* 2011). Most genetic studies published from the region either contain little intraspecific sampling (phylogenetic studies; Keogh *et al.* 1998; Osborne & Christidis 2001; Rawlings *et al.* 2004; Wuster *et al.* 2005; Norman *et al.* 2007) and/or limited geographical sampling from within New Guinea (De Bruyn *et al.* 2004; Baker *et al.* 2008; Cook & Hughes 2010; Craft *et al.* 2010). This may be due to the fact that many species in New Guinea have narrow ranges (Taylor *et al.* 1982; Allison 2007), making the inference of broadscale patterns of intraspecific genetic structure in the Australo-Papuan region difficult.

Previous studies have identified areas of endemism in the southwest Pacific, based on the distribution of various taxa (Van Welzen 1989; de Boer & Duffels 1996). Within New Guinea, major areas of endemism identified include, southern New Guinea, the Papuan Peninsula and northern New Guinea. Additionally, the Bismarck Archipelago, the Solomon Islands and islands of Vanuatu each possess unique species (de Boer & Duffels 1996). Biogeographical features that have led to vicariance have also been identified based on genetic relationships. These include the following: (i) the Carpentaria Gap, an ecological–climatic barrier separating the Northern Territory and Queensland (Bowman *et al.* 2010), (ii) the Torres Strait, the body of water currently separating Australia and New Guinea (Kearns *et al.* 2011) and (iii) the Central cordillera of New Guinea (Rawlings & Donnellan 2003; Kearns *et al.* 2011).

### Study system

**Fig. 1:**
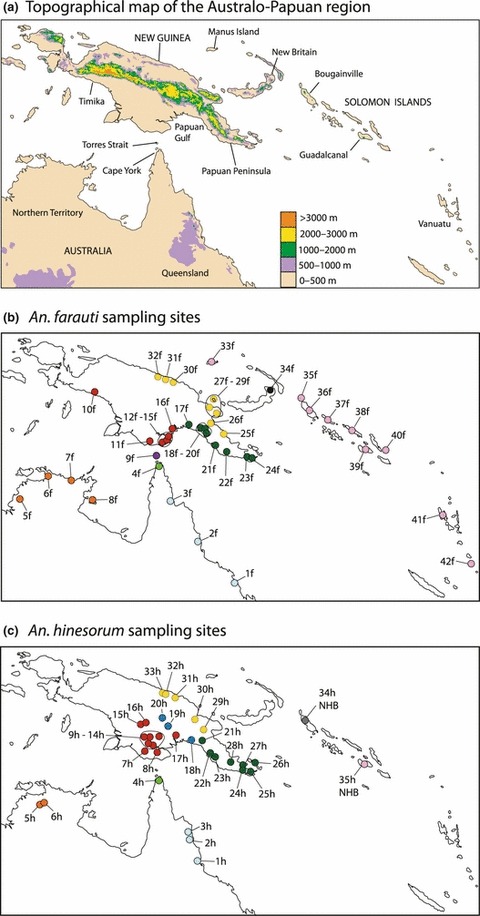
Maps of the southwest Pacific, including the topography of the region and approximate sampling locations for *Anopheles farauti* and *An. hinesorum*. Sites are colour coded by region in the same way throughout the study. See Fig 5 for key and Tables 1 and 2 for detailed sampling information.

In this study, we focus on three closely related mosquito species: *Anopheles farauti* (formerly *An. farauti* 1), *An. hinesorum* (formerly *An. farauti* 2) and *An. irenicus* (formerly *An. farauti* 7; Beebe *et al.* 2000c). Although morphologically indistinguishable, these species are identifiable using allozymes (Foley *et al.* 1993), PCR-based diagnostics and genomic DNA probes (Cooper *et al.* 1991; Beebe & Saul 1995) and are part of a larger species complex. Phylogenetic studies of the species complex suggest that the species under investigation are each other’s closest relatives (with the inclusion of the highland restricted species, *An. farauti* 5 and 6, which are most closely related to *An. hinesorum* and are only found in the Central Highlands of New Guinea; Foley *et al.* 1998; Beebe *et al.* 2000c; Beebe & Cooper 2002). Two of the species (*An. farauti* and *An. hinesorum*) are widespread malaria transmitting mosquitoes (Cooper *et al.* 2009), which occur east of the Webber line through New Guinea, the Bismarck Archipelago and the Solomon Islands with *An. farauti* extending to Vanuatu. The range of both species also extends southward into northern Australia, with *An. farauti* also present on the Torres Strait Islands between Australian and New Guinea (see Fig. 1a for a map of the region; Beebe & Cooper 2002; Cooper *et al.* 2002). In contrast *An. irenicus* has a very narrow range, limited to Guadalcanal in the Solomon Islands, and it does not bite humans (Beebe & Cooper 2002; Cooper *et al.* 2002). The species also differ in distribution and ecology; *An. farauti* is a coastally restricted mosquito (Beebe & Cooper 2002; Cooper *et al.* 2002) with larvae that are tolerant of brackish water (Sweeney 1987)—as are the larvae of *An. irenicus* (Foley & Bryan 2000). On the other hand, *An. hinesorum* is found both on the coast and inland, as well as in the cooler climate of New Guinea’s highlands (up to 1500 m; Cooper *et al.* 2002), and its larvae develop in freshwater. Despite this difference in larval salt tolerance, *An. farauti* and *An. hinesorum* are frequently found cohabiting freshwater coastal larval sites (Cooper *et al.* 2002). A better understanding of the population genetics and evolutionary relationships of these mosquitoes is of significant value in terms of malaria control, particularly with regards to the evolution and potential spread of insecticide-resistant (biochemical or behavioural) phenotypes.

Both *An. hinesorum* and *An. farauti* bite humans and mammals, as do most other species in the broader group of closely related cryptic species (the Punctulatus group; Cooper *et al.* 2009). Additionally, *An. koliensis* and *An. punctulatus* are positioned basally within the Punctulatus group (Beebe *et al.* 2000c) and are generalist feeders that bite humans and transmit malaria in the region (Cooper *et al.* 2009). Thus, the (generalist) ability to perceive humans as suitable hosts is most likely the ancestral character of the group. With this in mind, an interesting behavioural pattern has been observed—two populations belonging to different species co-occurr in the Solomon Islands and do not bite humans. These are *An. irenicus* (found only on the island of Guadalcanal) and the Solomon Island populations of *An*. *hinesorum* (Foley *et al.* 1994; Beebe *et al.* 2000a; Cooper & Frances 2002). Although limited to few individuals per species and mostly to single markers, previous phylogenetic studies suggest that *An. irenicus* is more closely related to *An. farauti* than to *An. hinesorum* (Foley *et al.* 1998; Beebe *et al.* 1999, 2000c, d; Bower *et al.* 2009). These relationships indicate that the ability to perceive humans as hosts may have been lost in co-occurring island populations of *An. irenicus* and *An. hinesorum*. If this is the case, there may be opportunities to investigate the genetic mechanisms involved in this loss, which would be of significant medical interest. Throughout the rest of manuscript, we refer to this loss of perception of humans as hosts as ‘non-human biting behaviour’. In contrast to *An. hinesorum* and *An. irenicus*, *An. farauti* bite humans and transmit malaria wherever there are infected hosts, including on the Solomon Islands.

The history of colonization of *An. farauti* into the Solomon Islands remains unclear and has only been investigated using ribosomal DNA ITS genotyping methods (Beebe *et al.* 2000b; Cooper *et al.* 2002) or limited geographical sampling (Hasan *et al.* 2008). Further, the history of colonization by *An. hinesorum* of the Solomon Islands has not yet been investigated. However, previous studies on *An. farauti* in the southwest Pacific have found significant regional population structure, both within and between landmasses, based on multiple ribosomal DNA internal transcribed spacer (rDNA ITS1 and ITS2) genotypes (Beebe *et al.* 2000b; Bower *et al.* 2008).

In this study, we aim to further examine evolutionary relationships between *An. farauti, An. hinesorum* and *An. irenicus* to explicitly assess whether human biting ability has been repeatedly lost in the Solomon Islands. We hypothesize that multiple gene trees will support the existence of discretely evolving lineages corresponding to recognized species and that *An. irenicus* will be most closely related to *An. farauti*, with *An. hinesorum* being more distantly related. Therefore, we also hypothesize that non-human biting behavior has evolved more than once in the species complex.

Additionally, we aim to compare geographical patterns of genetic differentiation between populations of *An. farauti* and *An. hinesorum* occurring in the Australo-Papuan region and islands of the southwest Pacific. We hypothesize that mitochondrial and nuclear sequence data will reveal population structure within New Guinea and Australia, as well as between the landmasses. As *An. hinesorum* is most commonly found inland, it is likely to encounter more barriers to dispersal than *An. farauti,* which inhabits a comparatively uniform coastal habitat. Thus, we predict that within New Guinea, *An. hinesorum* will contain more population structure than *An. farauti*. Sequence data for the mitochondrial cytochrome oxidase 1 (COI) gene, the single copy nuclear locus ribosomal protein S9 (rpS9) and cloned ITS2 sequence data were used to address these aims. This study design has allowed us to make comparisons between species, as well as between unlinked markers with very different modes of evolution.

## Materials and methods

### Sampling and laboratory procedures

Most mosquitoes were field collected from northern Australia, New Guinea, the Solomon Islands and Vanuatu by RD Cooper from the Australian Army Malaria Unit between 1991 and 2009. A few collection sites in Queensland were obtained through colleagues at Queensland Health. Significantly, this sampling encompasses almost the entire known geographical range of all species included in this study. Adult mosquitoes were caught either in CO_2_ light traps or by night human biting catches. Some specimens were also collected as larvae and reared to adulthood. Mosquitoes were identified by species-specific DNA probe and/or PCR–RFLP analysis (Cooper *et al.* 1991; Beebe *et al.* 1994; Beebe & Saul 1995), and those identified as *Anopheles farauti*, *An. hinesorum* and *An. irenicus* were used in this study. It should be noted that we do not have a complete (multilocus) set of data for all individuals. Sampling information on mosquito material (*An. farauti* and *An. hinesorum*) used is summarized in Tables 1–3 and sampling locations are shown in Fig. 1. For *An. irenicus*, all individuals used were sampled from Guadalcanal Island (one individual for ITS2, four individuals for COI and three individuals for rpS9).

**Table 1:** Sampling data and genetic diversity indices for *Anopheles farauti*

				COI			rpS9		
					
Region	Site ID*	Co-ordinates^†^	*n* (COI, rpS9)^‡^	Hd^§^	π^¶^	TD**	Hd^§^	π^¶^	TD**
Queensland	1f	−21.133, 149.183	2, 0	1	0.57	n/a	—	—	—
	2f	−17.691, 146.107	10, 0	0.956	0.61	0.03	—	—	—
	3f	−14.086, 143.676	5, 0	0.9	0.38	0.27	—	—	—
Cape York	4f	−10.778, 142.415	5, 0	0.8	0.99	1.45	—	—	—
Northern Territory	5f	−13.926, 130.482	3, 0	0	0	n/a	—	—	—
	6f	−11.8, 132.633	5, 0	0.9	0.49	−0.67	—	—	—
	7f	−12.125, 134.746	9, 0	0.417	0.21	−1.68	—	—	—
	8f	−13.841, 136.493	3, 0	0.667	0.13	n/a	—	—	—
Torres Strait	9f	−10.167, 142.2	10, 0	0.8	0.46	−0.09	—	—	—
Timika	10f	−4.835, 136.758^††^	5, 5	0.6	0.11	1.22	0.867	0.50	−1.16
Southern New Guinea	11f	−9.2, 141.583	10, 5	0.511	0.34	0.07	0.867	0.30	−0.4
	12f	−9.25, 142.768	0, 1	—	—	—	—	—	n/a
	13f	−9.07, 143.211	0, 10	—	—	—	0.632	0.20	−0.77
	14f	−8.938, 143.409	0, 3	—	—	—	0.733	0.21	n/a
	15f	−8.6, 143.557	0, 5	—	—	—	0.778	0.44	−0.58
	16f	−8.043, 143.723	10, 0	0.844	0.43	−0.36	—	—	—
	17f	−7.777, 144.889	13, 6	0.872	1.01	−1.36	0.939	1.42	1.2
Southern Papuan Peninsula	18f	−8.072, 146.04	9, 6	0.944	0.69	−0.09	0.909	1.25	0.54
	19f	−8.339, 146.549	6, 0	0.933	0.44	−1.39	—	—	—
	20f	−8.724, 146.569	0, 4	—	—	—	0.786	1.20	n/a
	21f	−9.491, 147.293	9, 0	0.944	0.84	0.46	—	—	—
	22f	−10.15, 148.368	0, 5	—	—	—	0.867	1.32	0.16
	23f	−10.438, 149.891	9, 7	0.833	0.36	−1.19	0.813	0.53	−1.43
	24f	−10.347, 150.564	9, 0	0.889	0.27	−0.91	—	—	—
Northern Papuan Peninsula	25f	−8.148, 148.137	5, 8	0.700	0.27	−0.17	0.742	0.26	−0.34
	26f	−7.123, 147.041	9, 9	0.806	0.45	−0.32	0.660	0.24	−0.47
	27f	−6.741, 147.535	0, 5	—	—	—	0.733	0.34	1.15
	28f	−5.959, 147.079	0, 3	—	—	—	0.8	0.32	n/a
	29f	−5.268, 147.178	0, 8	—	—	—	0.242	0.06	−1.5
Northern New Guinea	30f	−4.056, 144.741	9, 12	0.972	0.54	−0.17	0.663	0.22	−1.29
	31f	−3.865, 144.064	9, 6	0.944	0.47	−0.68	0.758	0.32	−0.72
	32f	−3.531, 143.589	3, 8	—	—	—	0.783	0.30	0.14
Manus	33f	Manus Island	5, 5	0.800	0.26	−0.17	0	0	0
New Britain	34f	−4.237, 152.15^††^	1, 0	n/a	n/a	n/a	—	—	—
Solomon Islands	35f	Buka Island	3, 0	0	0	n/a	—	—	—
	36f	Bougainville Island	14, 10	0.538	0.31	2.18	0.279	0.07	−1.14
	37f	Choiseul Island	10, 4	0.756	0.31	−0.33	0.464	0.12	n/a
	38f	Santa Isabel Island	0, 20	—	—	—	0.677	0.22	−0.6
	39f	Guadalcanal	9, 0	0.639	0.15	0.19	—	—	—
	40f	Ulawa Island	10, 5	0.733	0.19	−0.13	0.467	0.11	0.82
Vanuatu Islands	41f	Espiritu Santo	4, 0	0.833	0.41	n/a	—	—	—
	42f	Tanna Island	10, 0	0	0	0	—	—	—
Total Data	n/a	n/a	233, 160	0.976	2.20	0.44	0.766	0.98	−0.978

Data shown include sampling information (*site ID and ^†^geographic co-ordinates, ^‡^number of individuals (*n*) sequenced per locus), as well as genetic diversity indices for each locus [^§^haplotype diversity (Hd), ^¶^nucleotide diversity (π) and **neutrality tests (Tajima’s *D*)] per site and for total species data. Locations for islands are given as island names. ^††^Approximate co-ordinates. GenBank Accession numbers (COI: JN384217–JN384661; rpS9: JN384662–JN385275; ITS2: JN564766–JN564794, AF033218, HM584426, AF1404314–AF104326).

**Table 2:** Sampling data and genetic diversity indices for *Anopheles hinesorum*

				COI			rpS9		
					
Region	Site ID*	Co-ordinates^†^	*n* (COI, S9)^‡^	Hd^§^	π^¶^	TD**	Hd^§^	π^¶^	TD**
Queensland	1h	−18.216, 145.854	8, 0	0.929	0.50	0.69	—	—	—
	2h	−16.673, 145.328	6, 0	0.867	0.56	−0.25	—	—	—
	3h	−16.252, 145.302	7,0	0.952	0.49	−1.59	—	—	—
Cape York	4h	−10.954, 142.462	19, 0	0.965	1.19	−0.74	—	—	—
Northern Territory	5h	−12.96, 132.56	7, 0	0.810	0.23	0.05	—	—	—
	6h	−12.76, 132.66	4, 0	1	1.55	n/a	—	—	—
Southern New Guinea	7h	−8.634, 142.218	1, 2	—	—	—	1	1.51	n/a
	8h	−8.621, 141.137	3, 0	0	0	n/a	—	—	—
	9h	−8.074, 141.750	5, 2	0.900	0.91	−0.34	0	0	n/a
	10h	−7.728, 141.490	9, 6	0.889	1.22	0.46	0.939	1.31	0.58
	11h	−6.978, 141.503	0, 2	—	—	—	0.833	0.35	n/a
	12h	−7.182, 141.247	5, 9	0.900	0.64	−0.81	0.961	0.90	−1.01
	13h	−7.079, 141.989	6, 7	1	1.15	−0.06	0.571	0.83	n/a
	14h	−6.823, 141.371	10, 8	0.844	0.25	−1.04	0.575	0.43	0.73
	15h	−6.317, 141.024	0, 1	—	—	—	1	0.70	n/a
	16h	−6.121, 141.294	0, 2	—	—	—	0.833	0.70	n/a
	17h	−7.319, 144.178	0, 2	—	—	—	0.500	0.46	n/a
Central New Guinea	18h	−7.864, 145.669	11, 8	0.182	0.45	−2.05	0.342	0.09	−1.04
	19h	−6.78, 143.469	11, 12	0.618	0.23	−1.57	0.471	0.13	0.06
	20h	−6.205, 143.01	1, 10	—	—	—	0.584	0.18	0.87
Papuan Peninsula	21h	−7.966, 146.214	8, 9	0.964	0.47	−0.35	0.562	0.17	−1.53
	22h	−8.999, 146.801	9, 12	0.972	0.50	−0.97	0.308	0.08	−1.49
	23h	−9.145, 147.166	0, 1	—	—	—	0	0	n/a
	24h	−10.203, 149.695	0, 1	—	—	—	0	0	n/a
	25h	−10.329, 150.271	4, 3	1	0.69	n/a	0.533	0.12	n/a
	26h	−9.668, 150.787	12, 12	0.894	0.51	−0.37	0.163	0.04	−1.51
	27h	−9.704, 149.652	0, 2	—	—	—	0	0	0
	28h	−9.505, 148.462	4, 0	1	0.51	n/a	—	—	—
Northern New Guinea	29h	−6.86, 146.351	4, 3	0.833	0.38	−0.78	0.600	0.45	n/a
	30h	−5.769, 145.592	1, 0	n/a	n/a	n/a	—	—	—
	31h	−3.956, 143.927	1, 0	n/a	n/a	n/a	—	—	—
	32h	−3.782, 143.336	3, 3	0.667	0.38	0	0	0	0
	33h	−3.8, 143.066	5, 4	0.400	0.23	−1.05	0.786	0.39	−1.28
Solomon Islands	34h	Bougainville Island	21, 8	0.271	0.43	−2.45*	0	0	0
	35h	Guadalcanal	21, 11	0.348	0.80	−1.29	0.416	0.39	1.44
Total Data	n/a	n/a	206, 140	0.969	2.54	−0.343	0.872	1.09	−1.166

Data shown include sampling information (*site ID and ^†^geographic co-ordinates, ^‡^number of individuals (*n*) sequenced per locus), as well as genetic diversity indices for each locus [^§^haplotype diversity (Hd), ^¶^nucleotide diversity (π) and **neutrality tests (Tajima’s *D*)] per site and for total species data. Locations for islands are given as island names. Statistically significant results following Bonferroni correction with an asterisk. GenBank Accession numbers (COI: JN384217–JN384661; rpS9: JN384662–JN385275; ITS2: JN564766–JN564794, AF033218, HM584426, AF1404314–AF104326).

**Table 3:** Summary of *An. hinesorum* ITS2 sequencing

Region	Site ID	*n*	ITS2 sequences*
Queensland	3h	3†	2–4
Cape York	4h	3†	5–7
Northern Territory	5h	3†	1, 2
	6h	3†	1, 2
Southern New Guinea	8h	3†	8–9, 12–14
	9h	3†	2, 11–15
Central New Guinea	18h	4	16, 20
	19h	5	20
	20h	5	20
Papuan Peninsula	21h	3†	17–19
	22h	2†	17, 18
	25h	3†	17–19
	28h	3†	17, 18
Northern New Guinea	29h	4	21
	31h	1	21
	32h	1	21
	33h	3	21
Bougainville Island	34h	3‡	22–26
Guadalcanal Island	35h	3‡	27–29

* ITS2 sequences 1–29 correspond to GenBank Accession nos JN564766–JN564794.

† Three individuals per site were cloned with 3–5 random clones sequenced.

‡ Mosquito isolates from three separate larval sites.

#### DNA extraction, amplification, cloning and sequencing

Genomic DNA was extracted from all specimens using a standard salt extraction method (Beebe *et al.* 1999). Final PCR mixtures for all loci amplified contained 2.5 mm MgCl, 0.125 mm of each dNTP, 0.4 μm of each primer, 0.5–1.0 unit of *Taq* polymerase and 5.0–10.0 ng of extracted genomic DNA (1 μL of extraction). The cycling for all loci involved an initial denaturation step of 95 °C for 3 min, followed by 35 cycles of 95 °C for 1 min, 50 °C for 1 min (57 °C for rpS9) and 72 °C for 1 min. Part of the mtDNA COI gene was amplified using primers from Walton *et al.* (2000) and a novel reverse primer (5′-TAA TAT AGC ATA AAT TAT TCC-3′) yielding a 527 base pair (bp) product after editing. An exon priming intron crossing (EPIC) fragment of the nuclear-ribosomal protein S9 (rpS9) was amplified using the forward primer (5′-GAA AAG CCR CGT CTC GAT GCG G-3′) and the reverse primer (5′-GCC AAT CCC AGC TTG AAS ACC-3′), designed by aligning an *Aedes aegypti* mRNA sequence (GenBank: XM_001653919) to the *An. gambiae* genome, yielding a final length of 431 bp after editing. The ITS2 locus was amplified and cloned following procedures outlined in a previous study (Beebe *et al.* 1999). Amplified products were purified using the QIAquick PCR purification kit (Qiagen, Hilden, Germany) or using an enzyme digestion containing 1 unit of Exonuclease I, 1 unit of Antarctic Phosphatase and a final concentration of 1× Antarctic Phosphatase buffer per reaction, with an incubation period of 37 °C for 30 min followed by 80 °C for 5 min. Sequencing was performed by the Australian Genome Research Facility and by Macrogen (Korea) and sequences have been submitted to GenBank (refer to Table 1 for accession numbers).

The ITS2 is part of the rDNA repetitive gene family, and intragenomic copy variants of ITS2 frequently occur in *Anopheles* mosquitoes, preventing direct DNA sequencing. The ITS2 sequence data of *An. farauti* (GenBank numbers AF104314–AF104326) and *An. irenicus* (GenBank number AF033218) used in this study was generated in previous studies (Beebe *et al.* 1999), with geographically selected individuals of *An. farauti* PCR amplified, cloned and sequenced. New ITS2 sequences for *An. hinesorum* were generated for this study via PCR and cloning, from a subset of individuals representing as best as possible the distribution of this species. Approximately three *An. hinesorum* individuals per site were cloned and 3–5 randomly selected colonies sequenced following methods of (Beebe *et al.* 1999). Table 3 provides a summary of ITS2 sequencing of *An. hinesorum* individuals.

#### Sequence quality and editing

Prior to analysis, sequences were verified as being of mosquito origin by performing a blast search against the NCBI GenBank nucleotide database. For quality assurance, we translated all mtDNA COI nucleotide sequences into amino acid sequences and found no stop codons in the reading frame of any sequence. Sequences were aligned and edited in the program Geneious (Drummond *et al.* 2010), and pseudo-haplotypes of the nuclear locus rpS9 were inferred using the program PHASE, implemented in DNAsp v5 (Rozas *et al.* 2003). We tested for recombination within the nuclear loci using the program RPD3 (Martin *et al.* 2010).

### Data analysis

#### Genetic diversity and tests of neutrality

Tajima’s *D* (TD) test (Tajima 1989) was used to test for selective neutrality and equilibrium expectations and was computed for sites with five or more individuals sampled, as well as for the total data of each species in Arlequin v 3.5 (Excoffier & Lischer 2010). Bonferroni corrections were applied to assess the significance of TD tests. To estimate levels of genetic diversity, Arlequin was used to compute π (nucleotide diversity), and haplotype diversity (Hd) was calculated in DNAsp.

#### Phylogenetic and coalescent analyses

To infer evolutionary relationships between the species at mtDNA and nuclear DNA (nDNA) loci, phylogenies were constructed using Bayesian (beast; Drummond & Rambaut 2007) and maximum likelihood (ML) methods (PhyML; Guindon *et al.* 2009). For ML phylogenies, duplicate sequences (individuals with identical sequences) were removed from alignments prior to analysis, whereas for beast (coalescent) analyses, all sampled haplotypes were included. jModelTest version 0.1.1 (Posada 2008) was run using three substitution schemes to determine the most appropriate model of evolution to use in subsequent analyses. An HKY model (Hasegawa *et al.* 1985) with a gamma distribution and a proportion of invariable sites was used in analyses of the COI locus. For the rpS9 locus, a K80 (K2P) model (Kimura 1980) with a gamma distribution was used, and for the ITS2 locus, a GTR model with a gamma distribution was used. For all ML analyses, PhyML was run using the ML method with 1000 bootstraps. ITS2 data were only used for phylogenetic analysis because it is part of the multicopy rDNA gene family that evolves through a poorly described non-Mendelian process (Beebe *et al.* 1999; Alquezar *et al.* 2010). Additionally, *Anopheles* mosquitoes often carry multiple ITS2 sequence variants in the rDNA array, thus cloning is required prior to sequencing.

Analyses were also run in *beast (Heled & Drummond 2010) to estimate species/population relationships based on multilocus data and to estimate dates of divergence between *An. farauti* and *An. hinesorum*. Individuals were assigned to populations and species (as traits) in *beast analyses based on their geographic origin. Groups assigned for *An. farauti* include: southern New Guinea (sites 10–16f), Papuan Peninsula (sites 17–24f), northern New Guinea (sites 25–32f), Manus Island (site 33f) and Solomon Islands (sites 35–40f); groups for *An. hinesorum* include: southern New Guinea (sites 8–20h), Papuan Peninsula (sites 21–26h), northern New Guinea (sites 29–33h), north Solomon Islands (site 34h) and south Solomon Islands (site 35h). *An. irenicus* and the outgroup *An. koliensis* were also included in the *beast analysis. We specified a rate of 0.0115 mutations (2.3%) per million years for the COI locus, based on the widely used mitochondrial insect molecular clock (Brower 1994) and used the relaxed lognormal clock as recommended by the developers of beast as it allows the mutation rate to vary between branches (Drummond *et al.* 2006). We allowed the program to estimate the substitution rate of the rpS9 locus. beast was also run using the simpler strict clock model, but we found that the relaxed lognormal clock model outperformed the strict clock model. This was based on an estimation of Bayes factors between models: the log10 BF of the relaxed clock relative to the strict clock model = 0.667, which is taken as evidence in favour of a relaxed clock model (Kass & Raftery 1995). Bayes Factors were computed in Tracerv.1.5 (Rambaut & Drummond 2009) from the mean likelihoods of each model.

Two independent analyses of 50 million generations were run in BEAST and *BEAST, with trees sampled every 1000 generations. Log files and tree files from the two runs were then combined in LogCombiner v1.6.1 and a burnin of 10 million generations was applied. We assessed the performance of the combined runs in Tracer v1.5, and all ESS values were 200 or above for beast analyses, as recommended by the developers, however, for the *beast analyses, a few ESS values failed to reach 200 despite the long run time. Tree files generated from the programs were run in Tree Annotator version v1.6.1 and viewed in FigTree v1.3.1 (Rambaut 2009). We also performed parsimony based ancestral state reconstruction on the mitochondrial ML tree using the program Mesquite (Maddison & Maddison 2011). Where citations are not included, the above-mentioned programs are codistributed with beast.

#### Phylogeography and population structure

To examine phylogeographic relationships in *An. farauti* and *An. hinesorum*, we constructed maximum parsimony haplotype networks (using all sampled haplotypes) in tcs (Clement *et al.* 2000). Levels of differentiation between sites for COI (mtDNA) and rpS9 (nDNA) were estimated by generating pairwise *F*_ST_ values in Arlequin version 3.5 (distance method). The significance levels of *F*_ST_ comparisons were assessed using permutation tests (1023 permutations per comparison), also implemented in Arlequin. We only performed pairwise *F*_ST_ comparisons for populations with five or more individuals sampled. Due to the large number of sites sampled, we summarized pairwise *F*_ST_ relationships using multidimensional scaling plots generated in the program R version 1.4 (R Development Core Team 2011). Multidimensional scaling plots are similar to principle component analysis plots but relative distances between points represent relationships between pairwise comparisons. This allows a visually intuitive and compact representation of *F*_ST_-based relationships between sites (and the regions they were sampled from). Detailed tables summarizing *F*_ST_ relationships and significance of comparisons are available as supplementary material (Tables S1–S4, Supporting information).

## Results

### Genetic diversity and tests of neutrality/recombination

Tables 1 and 2 show genetic diversity indices and results of TD tests. Our data for *Anopheles farauti* contained a total of 82 COI haplotypes (Hd = 0.976, π = 2.20) and 49 (inferred) rpS9 haplotypes (Hd = 0.766, π = 0.98; Table 1). For *An. hinesorum*, we found a total of 94 COI haplotypes (Hd = 0.969, π = 2.54) and 45 (inferred) rpS9 haplotypes (Hd = 0.872, π = 1.09; Table 2). Most tests of selection gave nonsignificant results, including tests performed with the total data of each species at both loci. This suggests that populations approximate the expectations of mutation-drift equilibrium. The only site that gave a significant TD test value following Bonferroni correction was the *An. hinesorum* site 34h (TD = −2.45, *P* < 0.001). We found no evidence of recombination at either of the nuclear loci.

### Phylogenetic and coalescent analysis

**Fig. 2:**
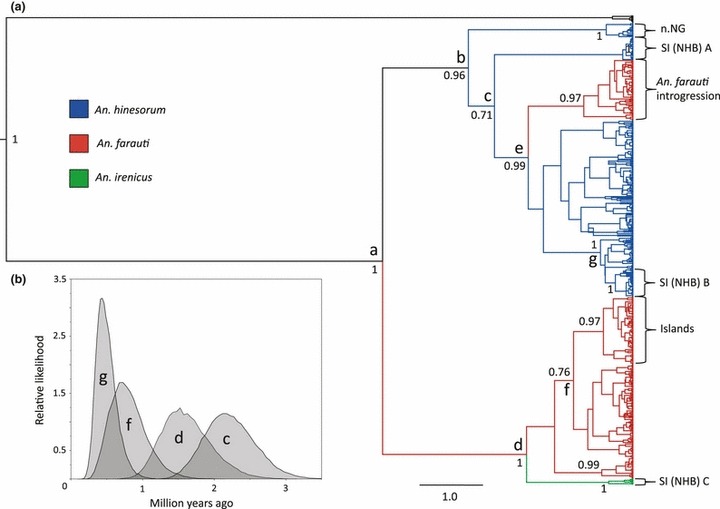
(a) Bayesian (beast) phylogeny (maximum clade credibility) of the COI locus containing all haplotypes sampled. Branches are coloured by species [red = *Anopheles farauti*, blue = *An. hinesorum*, green = *An. irenicus*, black = *An. koliensis* (outgroup)]. Support values are posterior probabilities, and scale bar shows time in millions of years. A standard insect molecular clock of 2.3% divergence per million years was used to scale the tree. Abbreviations used on labels include: NHB, non-human biting; SI, Solomon Islands. Further details on node and clade labels are described in the results section. (b) Graph generated from the combined beast log files showing the most likely dates of divergence of nodes on the beast phylogeny (a), as well as the range of dates divergence for nodes.

**Fig. 3:**
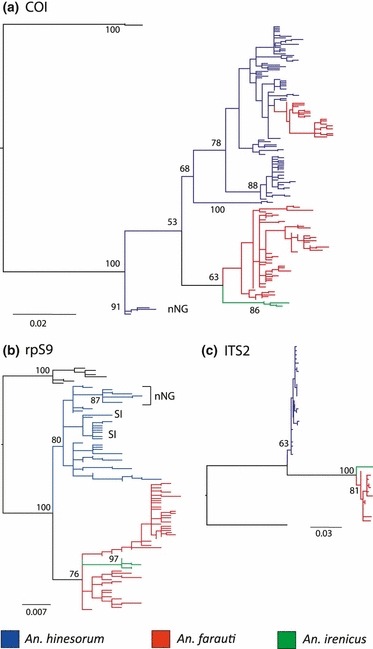
Maximum likelihood phylogenies of: (a) COI, (b) rpS9 and (c) ITS2. Duplicate sequences were removed prior to the generation of phylogenies. Branches are coloured by species [red = *Anopheles farauti*, blue = *An. hinesorum*, green = *An. irenicus*, black = *An. koliensis* (outgroup)]. Support values on nodes are percentages out of 1000 bootstraps; scale bar is in units of number of nucleotide substitutions per site, nNG, northern New Guinea.

Phylogenies of mitochondrial and nuclear markers (Figs 2a and 3) suggest that the non-human biting species, *An. irenicus*, is monophyletic and most closely related to the human biting *An. farauti*. On the basis of mitochondrial genealogies, we infer that four independent and strongly supported clades from the three species under investigation occur on the Solomon Islands (two *An. hinesorum*, and one of each of *An. farauti* and *An. irenicus*; Fig. 2a). Interestingly, the two (non-human biting) *An. hinesorum* Solomon Islands mtDNA lineages are phylogenetically distinct from each other. Ancestral state reconstruction supported human biting behaviour as the ancestral trait, and given the close relationship between *An. irenicus* and *An. farauti*, this supports the hypothesis that non-human biting behaviour has evolved at least twice on the Solomon Islands. Although monophyletic and distinct (with the exception of one Solomon Islands haplotype that is nested within a closely related clade sampled from the Papuan Peninsula), the less divergent lineage is most closely related to populations found on the Papuan Peninsula.

We found that *An. farauti* and *An. hinesorum* are polyphyletic for mtDNA, with a monophyletic lineage of *An. farauti* (found throughout Queensland, the Torres Strait Islands and southern New Guinea) nested within *An. hinesorum* (Figs 2a and 3a). In contrast to the mitochondrial trees, phylogenies of the two nuclear markers show that *An. farauti* and *An. hinesorum* are reciprocally monophyletic (Fig. 3b,c). This conflict between gene trees may be due to either ancient mitochondrial introgression, or incomplete lineage sorting at the mitochondrial locus.

In the mtDNA phylogenies, a basal lineage within *An. hinesorum* clearly stands out (∼3% divergence) and was sampled exclusively from northern New Guinea. However, there is conflict in the position of this clade between trees generated using different phylogenetic models. Maximum likelihood phylogenies suggest that it diverged earlier than the *An. hinesorum—An. farauti* split (Fig. 3a), whereas Bayesian methods suggest that it diverged after the two species split. In the rpS9 phylogeny, *An. hinesorum* from northern New Guinea also form a distinct clade, although unlike the mtDNA phylogenies populations from this region are nested within the rest of *An. hinesorum* (Fig. 3b). These populations are currently identified as *An. hinesorum* but their high level of divergence and genetic distinctness suggest that they may be a separate species.

**Fig. 4:**
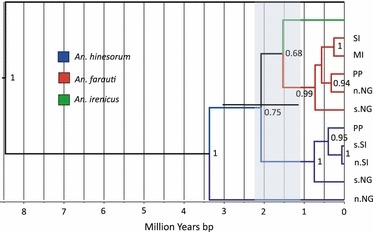
Phylogenetic relationships between species and populations estimated by jointly analysing COI and rpS9 loci in *beast. Branches are coloured by species as shown in the key and the outgroup is *Anopheles koliensis* (black). Support values are posterior probabilities, and the scale bar shows time in millions of years (based on the molecular clock used—2.3% per million years for mtDNA; nuclear DNA estimated by the program). The error bar on the node at which *An. farauti* and *An. hinesorum* split is the 95% HPD (height) and shows the error associated with the estimate of the timing of divergence between the species. The shaded area shows the 95% HPD (height) of the putative introgression event based on the mtDNA beast phylogeny (node e). Abbreviations include: SI, Solomon Islands; MI, Manus Island; PP, Papuan Peninsula; n.NG, northern New Guinea; s.NG, southern New Guinea; s.SI, southern Solomon Islands; and n.SI, northern Solomon Islands.

The multilocus tree generated using *beast (Fig. 4) reveals that *An. hinesorum* (with the exception of northern New Guinea) and *An. farauti* are reciprocally monophyletic. On the basis of the molecular clock used, it appears that the two species diverged about 2 Ma (95% HPD height: 3.04–1.15 Mya, Fig. 4). This confidence interval is overlapping with the estimated timing of the putative mitochondrial introgression (95% HPD height: 2.19–1.11 Mya, Fig. 3 node e and Fig. 4). Thus, the null hypothesis of ancestral polymorphism cannot be rejected as the cause of the mitochondrial polyphyly of *An. farauti*. The sister taxa relationship between *An. farauti* and *An. irenicus* is also supported by the multilocus tree, as is the early divergence (pre the split of *An. farauti* and *An. hinesorum*) of northern New Guinean populations of *An. hinesorum*. A close relationship can be seen between northern and southern Solomon Island populations in the multilocus tree. This can be explained by both mitochondrial lineages being found in the northern and the southern Solomon Islands, as well as there being heterozygotes between populations for the nuclear (rpS9) locus.

### Phylogeography and population structure

#### Anopheles farauti

**Fig. 5:**
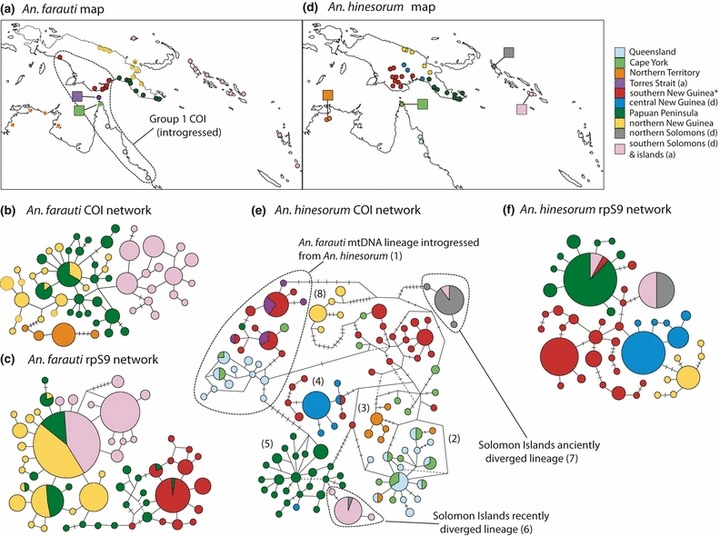
Maximum parsimony haplotype networks of *Anopheles farauti* and *An. hinesorum* at COI and rpS9 loci. Circles represent haplotypes and the size of circles is relative to the number of individuals per haplotype. Lines between haplotypes represent a single mutational change, as do dashes across lines, bends and intersections. Colours indicate sampling locality of haplotypes (i.e. geographic region), see maps and legend for details. The *An. farauti* lineage containing mtDNA introgressed from *An. hinesorum* is shown in the *An. hinesorum* network (Group 1 in e).

**Fig. 6:**
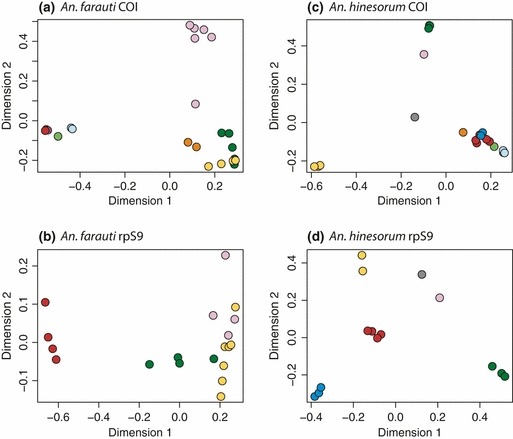
Multidimensional scaling plots of pairwise *F*_ST_ values. Each point represents a sampling site and distances between points are relative to *F*_ST_ values. Sites are coloured by geographical region, consistent with maps presented throughout.

The *An. farauti* mtDNA COI haplotype network (Fig. 5b), as well as MDS plots of pairwise *F*_ST_ comparisons (Fig. 6a), reveal well-defined geographical groups. Groups identified include the following: (1) Queensland—including Cape York; (2) Torres Strait, southern New Guinea and Timika (both of which are part of the *An. farauti* lineage containing mtDNA introgressed from *An. hinesorum* and are therefore shown on the *An. hinesorum* haplotype network: Fig. 5e, Group 1); (3) Northern Territory and (4) the Solomon and Vanuatu islands; (Figs 5b and 6a). Some structure is also evident between *An. farauti* populations from the southern Papuan Peninsula and northern New Guinea (Fig. 6a). Island populations of *An. farauti* form a monophyletic lineage (Fig. 5b). The most closely related mitochondrial relatives to the lineage with putatively introgressed mtDNA appear to be *An. hinesorum* from the Cape York Peninsula, which differ from the introgressed lineage by at least four mutational steps (Fig. 5e).

Though less informative, the rpS9 locus is largely concordant with the COI locus. The strongest genetic break at the rpS9 locus is between southern New Guinea and the other sites (Figs 5c and 6b). However, some specimens from the Papuan Peninsula have close affinities to the southern New Guinea populations, with two individuals (17f-15 and 17f-13) possessing a haplotype more commonly found in southern New Guinea (Fig. 5c). Although there are high fixation indices between some sites (Fig. 6b, Table S2, Supporting information), patterns of structure throughout the rest of New Guinea are difficult to distinguish.

#### Anopheles hinesorum

Both the haplotype network (Fig. 5e) and MDS plots of pairwise *F*_ST_ values (Fig. 6c) for the COI locus of *An. hinesorum* show that there is strong genetic structure between geographically distinct groups of this species. The haplotype network reveals that the Queensland and Cape York populations form a group (Fig. 5e: Group 2) that is closely related to a Northern Territory group (Fig. 5e: Group 3) and also to haplotypes from southern New Guinea. Within Group 2 are two haplotypes shared with individuals from the Northern Territory 3, possibly suggesting recent dispersal events from Queensland to the Northern Territory. Additionally, a number of individuals from Cape York do not fall within Group 2, but are more closely related to individuals from southern New Guinea with which they make up the highly reticulated centre of the network (Fig. 5e), although no haplotypes are shared between Cape York and southern New Guinea. Also closely related to the southern New Guinean populations are populations from central New Guinea (Fig. 5e: Group 4), and there are shared haplotypes between these regions. The MDS plot of pairwise *F*_ST_ values suggests a close affinity between all regions outlined previously (Fig. 6c), although sites from different regions are significantly differentiated from each other (Table S3, Supporting information).

Mitochondrial haplotypes of all *An. hinesorum* individuals from the Papuan Peninsula form a distinct group (Group 5) and MDS plots of pairwise *F*_ST_ values show that this region is very different from other regions sampled (Fig. 6c). Group 6 was sampled from the Solomon Islands (mostly Guadalcanal) and is nested within Group 5, suggesting a founder event from the Papuan Peninsula to Guadalcanal. Group 7 is another Solomon Island lineage (mostly from Bougainville) that is located at the opposite end of the network to Group 6 and is highly diverged from all groups. MDS plots show that sites from the Solomon Islands are highly distinct from each other and from all other regions. Finally, Group 8 is a highly differentiated lineage from northern New Guinea that differs from its closest relatives by at least 15 mutational steps (∼3%).

The *An. hinesorum* rpS9 locus revealed strong genetic structure, supported both by the haplotype network (Fig. 5f) as well as by MDS plots and pairwise *F*_ST_ values (Fig. 6d, Table S4, Supporting information). Distinct groups identified include the following: (i) southern New Guinea—forming the centre of the network; (ii) central New Guinea; and (iii) the Papuan Peninsula (Fig. 5f)—with one haplotype of seven found on the Papuan Peninsula being the most common. This common haplotype was also found on Guadalcanal (in the Solomon Islands) and there is a second more divergent Solomon Island haplotype found on both Bougainville and Guadalcanal. This finding supports the evidence from the mitochondrial data; that there are two genetically distinct *An. hinesorum* lineages inhabiting the Solomon Islands, with the less divergent lineage being most similar to populations from the Papuan Peninsula. Interestingly, some individuals from the Solomon Islands were found to be heterozygous for the two rpS9 alleles sampled from this region, suggesting that despite being highly diverged at the mitochondrial locus, individuals from the two putative founder events have remained reproductively compatible.

## Discussion

In this study, we examined evolutionary relationships of three closely related mosquito species at mtDNA and nDNA loci. We inferred that the non-human biting species *Anopheles irenicus* is most closely related to *An. farauti* and more distantly related to the non-human biting populations of *An. hinesorum*. Therefore, we conclude that non-human biting behaviour has evolved more than once in this group of cryptic species. Given the role of these mosquitoes in the transmission of malaria in this region, this is a medically significant finding.

Further, we provided the first genetic data on the colonization history of *An. hinesorum* to the Solomon Islands and found two distinct mitochondrial lineages of *An. hinesorum* on the Solomon Islands. We also found nonmonophyly between *An. hinesorum* and *An. farauti* at the mtDNA COI locus. Given that the species are monophyletic at nuclear loci, mtDNA introgression of *An. hinesorum* onto a nuclear background of *An. farauti* may have occurred; however, we cannot rule out incomplete lineage sorting as an alternative explanation. Finally, we showed that there are highly distinct populations of both *An*. *farauti* and *An. hinesorum* within and between New Guinea and Australia, with more strongly defined groups in *An. hinesorum*.

### Colonization of the Solomon Islands and the repeated evolution of non-human biting behaviour

We have presented evidence that four separate mtDNA lineages from the three species studied occur on the Solomon Islands (Fig. 2a): two lineages of *An. hinesorum* (one highly diverged and one less diverged), one lineage of *An. irenicus* and one lineage of *An. farauti*. The less divergent lineage of *An. hinesorum* on the Solomon Islands appears to be descended from a population on the Papuan Peninsula (Fig. 5e, group 6) that is known to bite humans and carry malaria and probably arrived on the Solomon Islands within the last 500 000 years. Due to the greater separation and recent tMRCA of the more anciently diverged Solomon Islands lineage of *An. hinesorum*, it is difficult to tell where it originated or when it diverged from the rest of the species. According to the haplotype networks, it is most closely related to populations from southern New Guinea (Fig. 5e, group 7) that bite humans and transmit malaria.

As previously mentioned, neither *An. hinesorum* nor *An. irenicus* bite humans in the Solomon Islands. On the basis of the phylogenetic evidence available from both mitochondrial and nuclear data, we conclude that this medically important behaviour has evolved more than once in these mosquitoes, possibly in the same geographical region. However, it is difficult to tell whether this behavior evolved more than once in *An. hinesorum*, because the two divergent mitochondrial lineages from the Solomon Islands appear to be reproductively compatible and may have exchanged this trait. On the basis of the geographical distribution of this trait, we think it most likely that non-human biting behaviour evolved in the Solomon Islands. We cannot, however, rule out the possibility that it evolved in New Guinea (where it may still be present in a subset of the population) or elsewhere and subsequently became fixed in the Solomon Islands through a founder event.

The finding of parallel evolution of non-human biting behaviour raises important evolutionary questions about the driving force behind the repeated evolution of this behaviour. We hypothesize that a reduction in host availability may have driven the parallel evolution of non-human biting behavior. Olfactory receptors (ORs) are a highly diverse gene family and it has recently been suggested that the number of OR genes possessed by a mosquito species is positively correlated with diversity of host preference (Arensburger *et al.* 2010). Rodents, bats and birds would have constituted the only warm-blooded hosts on the Solomon Islands at the times of initial colonization by *An. hinesorum* and *An. irenicus* (Carvajal & Adler 2005). Thus, it is possible that the colonizations of the Solomon Islands by *An. irenicus* and *An. hinesorum* resulted in a reduction in their OR gene repertoires due to relaxation of (purifying) selection on some OR genes upon exposure to a less diverse array of hosts. By genomic comparison of closely related populations with different host preferences, it may be possible to identify genes that enable mosquitoes to cue to humans. This would potentially reveal the mechanistic basis of attraction of *Anopheles* mosquitoes to humans, which may lead to the development of improved mosquito attractants and repellents, or other novel technologies to reduce direct exposure to infectious mosquitoes.

### Mitochondrial introgression and its potential role in range expansion

Mitochondrial introgression has been found by many studies across diverse groups (Ballard 2000; Funk & Omland 2003) and we suggest that mitochondrial introgression may have occurred between *An. hinesorum* and *An. farauti*. We base this on the finding that *An. farauti* is polyphyletic for mtDNA (COI), with a clade currently defined as *An. farauti* falling within *An. hinesorum* (Figs 2a and 3a), and the two species being monophyletic at nuclear loci (Fig. 3b,c). Although we cannot rule out incomplete lineage sorting as an explanation for this observation, we suggest that the observed polyphyly is probably to be the result of introgression. Because of its smaller effective population size, mtDNA tends to have a higher rate of genetic drift than nuclear DNA. Therefore, incomplete lineage sorting is more likely for nuclear than for mitochondrial loci. Additionally, all *An. farauti* individuals with introgressed mtDNA were sampled from an area geographically separate from the rest of the species, namely in Queensland, Torres Strait, southern New Guinea and Timika. These introgressed haplotypes are most closely related to populations of *An. hinesorum* in the same area (Fig. 5b, Queensland, Cape York and southern New Guinea). Overlapping distributions of species between which introgression is suspected is one of the keys to discriminating between mitochondrial introgression and incomplete lineage sorting (Funk & Omland 2003).

Mitochondria may frequently play an important role in thermal adaptation (Ballard & Whitlock 2004) and other studies have found evidence of temperature-based selection on mitochondria (Weinstein & Somero 1998; Doi *et al.* 1999). With this in mind, it is interesting to note that *An. hinesorum* occur at altitudes of >1000 m in the Central Ranges of New Guinea in colder conditions (avg. temp. 20 °C) than the coastally restricted *An. farauti* (avg. coastal temp. 26 °C; McAlpine *et al.* 1983). Additionally, the two sister species of *An. hinesorum*—*An. farauti* 5 and *An. farauti* 6—are only extant in the highlands of New Guinea (>1000 m; Cooper *et al.* 2002). Thus, a selective sweep may have driven an introgressed *An. hinesorum* mitotype, pre-adapted to cooler climates, through the southerly distribution of *An. farauti*, facilitating the spread of the species south (as this lineage occupies the species’ most southern latitude). Alternatively, rather than being favoured by selection, the introgressed mitochondrial lineage may have become fixed in populations during a range expansion. Fixation of introgressed genes and/or organelles following, or as a result of a range expansion, is an evolutionary phenomenon that has received recent support (Excoffier *et al.* 2009; Neiva *et al.* 2010).

### Patterns of divergence and genetic structure in New Guinea and northern Australia

Many of the genetically distinct groups identified by this study correlate with areas of endemism identified by previous biogeographic studies of insects and other taxa (see Introduction for a brief description of these areas; Van Welzen 1989; de Boer & Duffels 1996). We found phylogeographic patterns that are in agreement with these studies. For example, we found that populations of *An*. *farauti* and *An. hinesorum* from southern New Guinea are genetically similar to populations from Northern Queensland (and, in the case of *An. farauti*, to populations from Torres Strait). These relationships may be due to the fact that Australia and New Guinea were connected during most of the late Pleistocene (Voris 2000), and are similar to patterns observed in other species (Rawlings *et al.* 2004; Bower *et al.* 2008), although others have found that there has been little or no recent gene flow between the regions (Kearns *et al.* 2011). Also in agreement with previous studies of non-mosquito species was the strong differentiation between Northern Territory and Queensland populations (Bowman *et al.* 2010; Kearns *et al.* 2011). This may be due to the low rainfall area in the Gulf of Carpentaria (the Carpentaria Gap) that is hypothesized to have posed a barrier to dispersal for a number of species (Bowman *et al.* 2010). On the basis of mtDNA, *An. farauti* from the Northern Territory is not part of the introgressed *An. farauti* lineage and is most closely related to northern New Guinea populations. This pattern is different to those shown in previous nuclear-ribosomal-based studies of *An. farauti* which have suggested that there is some sharing of rDNA copy variant sequences between Queensland and the Northern Territory populations (Beebe *et al.* 2000b; Bower *et al.* 2008). This difference suggests that there may have been greater nuclear than mitochondrial gene exchange between Queensland and Northern Territory populations.

A number of geographically coherent groups of *An. hinesorum* within New Guinea were found to be almost completely genetically separate from each other. The greater distinction between groups of *An. hinesorum*, versus between groups of *An. farauti,* may be due to differences in behaviour or ecology. For example, there may be less distinction between *An. farauti* populations because of the relative uniformity of their coastal habitats and the absence of physical barriers to dispersal. The most highly diverged group that we found in either species within New Guinea was a group of *An. hinesorum* sampled from the Sepik and Ramu River regions in northern New Guinea (29–33h: Table 2, Fig. 1c). It is unsurprising to find such high levels of divergence between the north and south of New Guinea, as the Central Cordillera (up to 4000 m altitude) runs the length of the New Guinea and provides an obvious barrier to gene flow. Other studies have also found high levels of divergence between the north and south of New Guinea (Rawlings & Donnellan 2003; Kearns *et al.* 2011), although others have found no genetic structure between the regions (Murphy *et al.* 2007). The absence of shared nuclear rpS9 alleles between the northern New Guinean and other lineages, as well as the level of COI divergence (approximately 3%), and the fact that genomic DNA probes developed to identify *An. hinesorum* do not hybridize with samples of the species from this region (Cooper *et al.* 2002), indicate that *An. hinesorum* occupying northern New Guinea may be a separate species.

Some phylogeographic patterns that are congruent between the species and loci examined are not as easily explained as the divergence between populations from the north and south of New Guinea. One such example is the major genetic break in New Guinea, separating the Papuan Peninsula and southern New Guinea. This break is present at both mitochondrial and nuclear loci of both species (Figs 5 and 6). In *An. hinesorum*, the discontinuity occurs in the Gulf region of New Guinea, at which point three distinct groups are found in close proximity. This genetic break may be due to a mountain ridge of the Central Ranges that extends to approximately 20 km from the coast and separates the Purari River Basin from the Papuan Peninsula. The Purari River flows from Mt. Hagen in the Central Ranges to the ocean near site 18h (Fig. 1c) in the Gulf Province of PNG, offering an explanation for the close affinities observed between the lowland site (18h) and the sites sampled in the highlands (19 and 20h: Fig. 1c). Site 20h sits above 600 m in the Central Ranges and *An. hinesorum* has been collected from above 1300 m in this region (Cooper *et al.* 2002).

A similar genetic break between the Papuan Peninsula and southern New Guinea was also observed in *An. farauti* at both loci, but it occurs further to the west. Interestingly, this break correlates with a climatic interchange between monsoonal to the south and continuous hot wet to the north, suggesting that differences in rainfall or habitat associated with rainfall may have maintained the distinction between the regions (despite their close geographic proximity). This climatic interchange also delimits the group containing introgressed mtDNA from the group containing native *An. farauti* mtDNA. *An. farauti* rpS9 data revealed evidence of hybridization between populations from either side of the genetic break at a single site (17f: Figs 1b and 5c) but there is no evidence of these alleles elsewhere in the Papuan Peninsula.

This study provides important insights into the population genetic connectivity of two important malaria transmitting species in the southwest Pacific region. We find substantial population structure consistent with low gene flow both within and between major landmasses. This result implies that the spread of any newly evolved alleles conferring advantages against mosquito control initiatives, including biochemical and behavioural insecticide resistance, would be somewhat restricted in moving among populations relative to a high gene flow scenario (although the spread would also depend on the specific selection coefficient of a new allele). Genetic structure that cannot be explained by obvious barriers to dispersal, such as oceans and mountains, is apparent for both species.

## Data accessibility

DNA sequences: GenBank Accession nos

COI: JN384217–JN384661

rpS9: JN384662–JN385275

ITS2: JN564766–JN564794, AF033218, HM584426, AF104314–AF104326 (also see Table 1 and the supplementary spreadsheet).

## Supporting information

**Data S1.** Spreadsheet of Genbank Accession numbers with sample information for individuals used in this study.

**Data S2.** Spreadsheet of Genbank Accession numbers with sample information for individuals used in this study.

**Data S3.** Spreadsheet of Genbank Accession numbers with sample information for individuals used in this study.

**Table S1** Pair-wise *F*_ST_ values between sites for the *Anopheles farauti* COI locus.

**Table S2** Pair-wise *F*_ST_ values between sites for the *Anopheles farauti* rpS9 locus.

**Table S3** Pair-wise *F*_ST_ values between sites for the *Anopheles hinesorum* COI locus.

**Table S4** Pair-wise *F*_ST_ values between sites for the *Anopheles hinesorum* rpS9 locus.
