# Supplementary material for: Population structure, mitochondrial polyphyly and the repeated loss of human biting ability in anopheline mosquitoes from the southwest Pacific
Source: Mol Ecol. 2012 Sep;21(17):4327–43. doi: 10.1111/j.1365-294X.2012.05690.x (PMC3470930; doi:10.1111/j.1365-294X.2012.05690.x)
Supplement: Supplementary file 4 [file mec0021-4327-SD4.doc]

|  | **2f** | **3f** | **4f** | **6f** | **7f** | **9f** | **10f** | **11f** | **16f** | **17f** | **18f** | **19f** | **21f** | **23f** | **24f** | **25f** | **26f** | **30f** | **31f** | **33f** | **36f** | **37f** | **39f** | **40f** |
| --- | --- | --- | --- | --- | --- | --- | --- | --- | --- | --- | --- | --- | --- | --- | --- | --- | --- | --- | --- | --- | --- | --- | --- | --- |
| **3f** | **0*** |  |  |  |  |  |  |  |  |  |  |  |  |  |  |  |  |  |  |  |  |  |  |  |
| **4f** | **0.12*** | **0.12*** |  |  |  |  |  |  |  |  |  |  |  |  |  |  |  |  |  |  |  |  |  |  |
| **6f** | 0.87 | 0.9 | 0.82 |  |  |  |  |  |  |  |  |  |  |  |  |  |  |  |  |  |  |  |  |  |
| **7f** | 0.9 | 0.93 | 0.88 | **0*** |  |  |  |  |  |  |  |  |  |  |  |  |  |  |  |  |  |  |  |  |
| **9f** | 0.63 | 0.67 | 0.41 | 0.89 | 0.92 |  |  |  |  |  |  |  |  |  |  |  |  |  |  |  |  |  |  |  |
| **10f** | 0.69 | 0.8 | 0.5 | 0.93 | 0.96 | **0.1*** |  |  |  |  |  |  |  |  |  |  |  |  |  |  |  |  |  |  |
| **11f** | 0.68 | 0.73 | 0.49 | 0.91 | 0.93 | **0*** | **0.05*** |  |  |  |  |  |  |  |  |  |  |  |  |  |  |  |  |  |
| **16f** | 0.64 | 0.67 | 0.38 | 0.9 | 0.92 | **0*** | 0.3 | **0.11*** |  |  |  |  |  |  |  |  |  |  |  |  |  |  |  |  |
| **17f** | 0.78 | 0.78 | 0.73 | 0.48 | 0.57 | 0.78 | 0.8 | 0.8 | 0.78 |  |  |  |  |  |  |  |  |  |  |  |  |  |  |  |
| **18f** | 0.84 | 0.85 | 0.8 | 0.59 | 0.69 | 0.85 | 0.88 | 0.87 | 0.86 | **0*** |  |  |  |  |  |  |  |  |  |  |  |  |  |  |
| **19f** | 0.87 | 0.89 | 0.83 | 0.63 | 0.75 | 0.88 | 0.93 | 0.9 | 0.89 | **0.07*** | **0.05*** |  |  |  |  |  |  |  |  |  |  |  |  |  |
| **21f** | 0.83 | 0.84 | 0.78 | 0.57 | 0.67 | 0.84 | 0.86 | 0.86 | 0.85 | **0.06*** | **0*** | **0.11*** |  |  |  |  |  |  |  |  |  |  |  |  |
| **23f** | 0.87 | 0.9 | 0.84 | 0.69 | 0.77 | 0.89 | 0.93 | 0.91 | 0.89 | **0.01*** | **0.04*** | 0.21 | 0.17 |  |  |  |  |  |  |  |  |  |  |  |
| **24f** | 0.89 | 0.92 | 0.86 | 0.74 | 0.81 | 0.9 | 0.94 | 0.92 | 0.9 | **0.05*** | **0.07*** | 0.23 | 0.21 | **0*** |  |  |  |  |  |  |  |  |  |  |
| **25f** | 0.88 | 0.92 | 0.83 | 0.73 | 0.83 | 0.89 | 0.95 | 0.91 | 0.89 | 0.35 | 0.5 | 0.6 | 0.52 | 0.67 | 0.72 |  |  |  |  |  |  |  |  |  |
| **26f** | 0.86 | 0.89 | 0.82 | 0.66 | 0.75 | 0.87 | 0.91 | 0.89 | 0.87 | 0.25 | 0.36 | 0.42 | 0.42 | 0.49 | 0.53 | **0.02*** |  |  |  |  |  |  |  |  |
| **30f** | 0.85 | 0.87 | 0.81 | 0.62 | 0.72 | 0.86 | 0.9 | 0.88 | 0.86 | **0.02*** | **0.05*** | 0.17 | 0.21 | 0.12 | 0.16 | 0.39 | 0.18 |  |  |  |  |  |  |  |
| **31f** | 0.86 | 0.89 | 0.83 | 0.64 | 0.73 | 0.87 | 0.91 | 0.89 | 0.88 | **0*** | **0.02*** | **0.1*** | 0.18 | **0.03*** | **0.05*** | 0.51 | 0.32 | **0*** |  |  |  |  |  |  |
| **33f** | 0.9 | 0.93 | 0.87 | 0.77 | 0.86 | 0.91 | 0.96 | 0.93 | 0.92 | 0.51 | 0.64 | 0.72 | 0.59 | 0.76 | 0.79 | 0.84 | 0.75 | 0.68 | 0.69 |  |  |  |  |  |
| **36f** | 0.91 | 0.93 | 0.9 | 0.81 | 0.85 | 0.92 | 0.95 | 0.93 | 0.92 | 0.52 | 0.62 | 0.67 | 0.57 | 0.7 | 0.72 | 0.81 | 0.74 | 0.67 | 0.67 | 0.64 |  |  |  |  |
| **37f** | 0.91 | 0.93 | 0.89 | 0.82 | 0.87 | 0.91 | 0.95 | 0.93 | 0.92 | 0.56 | 0.66 | 0.72 | 0.61 | 0.75 | 0.77 | 0.83 | 0.77 | 0.71 | 0.72 | 0.74 | 0.19 |  |  |  |
| **39f** | 0.91 | 0.95 | 0.9 | 0.86 | 0.91 | 0.93 | 0.97 | 0.94 | 0.93 | 0.58 | 0.69 | 0.78 | 0.63 | 0.81 | 0.83 | 0.89 | 0.81 | 0.75 | 0.77 | 0.85 | 0.6 | 0.56 |  |  |
| **40f** | 0.91 | 0.94 | 0.9 | 0.85 | 0.9 | 0.92 | 0.96 | 0.94 | 0.92 | 0.61 | 0.71 | 0.78 | 0.65 | 0.81 | 0.83 | 0.88 | 0.81 | 0.76 | 0.77 | 0.81 | 0.53 | 0.5 | 0.29 |  |
| **42f** | 0.94 | 0.97 | 0.93 | 0.92 | 0.95 | 0.95 | 0.99 | 0.96 | 0.95 | 0.62 | 0.75 | 0.86 | 0.70 | 0.86 | 0.89 | 0.95 | 0.86 | 0.81 | 0.82 | 0.91 | 0.41 | 0.46 | 0.78 | 0.68 |

**Table S1** Pair-wise F_ST_ values between sites for the *Anopheles farauti* COI locus.

All sites are statistically differentiated (p<0.05) except for sites in bold text with asterisks. Significance tests are based on null distributions of haplotypes generated by 1023 permutations of haplotypes between each comparison of populations.

|  | **10f** | **11f** | **13f** | **15f** | **17f** | **18f** | **22f** | **23f** | **25f** | **26f** | **27f** | **29f** | **30f** | **31f** | **32f** | **33f** | **36f** | **38f** |
| --- | --- | --- | --- | --- | --- | --- | --- | --- | --- | --- | --- | --- | --- | --- | --- | --- | --- | --- |
| **11f** | **0*** |  |  |  |  |  |  |  |  |  |  |  |  |  |  |  |  |  |
| **13f** | **0.04*** | **0*** |  |  |  |  |  |  |  |  |  |  |  |  |  |  |  |  |
| **15f** | **0*** | **0*** | **0.02*** |  |  |  |  |  |  |  |  |  |  |  |  |  |  |  |
| **17f** | 0.35 | 0.42 | 0.52 | 0.39 |  |  |  |  |  |  |  |  |  |  |  |  |  |  |
| **18f** | 0.53 | 0.6 | 0.68 | 0.57 | **0*** |  |  |  |  |  |  |  |  |  |  |  |  |  |
| **22f** | 0.54 | 0.61 | 0.7 | 0.57 | **0*** | **0*** |  |  |  |  |  |  |  |  |  |  |  |  |
| **23f** | 0.77 | 0.81 | 0.85 | 0.79 | 0.21 | **0.07*** | **0.07*** |  |  |  |  |  |  |  |  |  |  |  |
| **25f** | 0.84 | 0.88 | 0.9 | 0.86 | 0.3 | 0.17 | 0.15 | 0.15 |  |  |  |  |  |  |  |  |  |  |
| **26f** | 0.85 | 0.89 | 0.91 | 0.87 | 0.32 | 0.17 | 0.16 | **0.05*** | **0.04*** |  |  |  |  |  |  |  |  |  |
| **27f** | 0.82 | 0.87 | 0.9 | 0.84 | 0.25 | **0.11*** | **0.1*** | **0.04*** | **0.1*** | **0*** |  |  |  |  |  |  |  |  |
| **29f** | 0.9 | 0.94 | 0.94 | 0.91 | 0.37 | 0.23 | 0.23 | 0.15 | 0.18 | 0.09 | 0.25 |  |  |  |  |  |  |  |
| **30f** | 0.87 | 0.9 | 0.91 | 0.88 | 0.38 | 0.21 | 0.21 | **0.01*** | 0.13 | **0.01*** | **0.06*** | 0.09 |  |  |  |  |  |  |
| **31f** | 0.83 | 0.88 | 0.9 | 0.85 | 0.31 | 0.17 | 0.18 | **0*** | 0.28 | 0.16 | 0.12 | 0.33 | **0.07*** |  |  |  |  |  |
| **32f** | 0.83 | 0.87 | 0.89 | 0.85 | 0.29 | 0.14 | 0.13 | **0.06*** | **0.02*** | **0*** | **0*** | 0.2 | **0.05*** | 0.14 |  |  |  |  |
| **33f** | 0.89 | 0.93 | 0.94 | 0.91 | 0.31 | 0.17 | 0.17 | 0.11 | 0.16 | **0.06*** | 0.22 | **0*** | **0.07*** | 0.3 | 0.17 |  |  |  |
| **36f** | 0.77 | 0.81 | 0.84 | 0.79 | 0.32 | 0.2 | 0.19 | 0.14 | 0.16 | 0.13 | 0.15 | 0.12 | 0.16 | 0.2 | 0.16 | **0.08*** |  |  |
| **38f** | 0.88 | 0.9 | 0.91 | 0.89 | 0.47 | 0.35 | 0.36 | 0.28 | 0.3 | 0.26 | 0.32 | 0.25 | 0.27 | 0.37 | 0.31 | 0.23 | 0.21 |  |
| **40f** | 0.86 | 0.91 | 0.92 | 0.88 | 0.27 | 0.15 | **0.14*** | 0.11 | 0.19 | 0.13 | 0.22 | 0.18 | 0.14 | 0.29 | 0.2 | **0.22*** | **0.11*** | 0.27 |

**Table S2** Pair-wise F_ST_ values between sites for the *Anopheles farauti* rpS9 locus.

All sites are statistically differentiated (p<0.05) except for sites in bold text with asterisks. Significance tests are based on null distributions of haplotypes generated by 1023 permutations of haplotypes between each comparison of populations.

**Table S3** Pair-wise F_ST_ values between sites for the *Anopheles hinesorum* COI locus.

|  | **1h** | **2h** | **3h** | **4h** | **5h** | **9h** | **10h** | **12h** | **13h** | **14h** | **18h** | **19h** | **21h** | **22h** | **26h** | **29h** | **32h** | **33h** | **34h** |
| --- | --- | --- | --- | --- | --- | --- | --- | --- | --- | --- | --- | --- | --- | --- | --- | --- | --- | --- | --- |
| **2h** | **0.05*** |  |  |  |  |  |  |  |  |  |  |  |  |  |  |  |  |  |  |
| **3h** | **0*** | **0.01*** |  |  |  |  |  |  |  |  |  |  |  |  |  |  |  |  |  |
| **4h** | **0.03*** | **0.07*** | **0.08*** |  |  |  |  |  |  |  |  |  |  |  |  |  |  |  |  |
| **5h** | 0.79 | 0.8 | 0.8 | 0.53 |  |  |  |  |  |  |  |  |  |  |  |  |  |  |  |
| **9h** | 0.56 | 0.55 | 0.57 | 0.25 | 0.72 |  |  |  |  |  |  |  |  |  |  |  |  |  |  |
| **10h** | 0.53 | 0.52 | 0.53 | 0.32 | 0.58 | **0.17*** |  |  |  |  |  |  |  |  |  |  |  |  |  |
| **12h** | 0.69 | 0.69 | 0.71 | 0.4 | 0.81 | **0.24*** | 0.3 |  |  |  |  |  |  |  |  |  |  |  |  |
| **13h** | 0.63 | 0.61 | 0.62 | 0.43 | 0.7 | 0.32 | 0.22 | 0.51 |  |  |  |  |  |  |  |  |  |  |  |
| **14h** | 0.8 | 0.81 | 0.81 | 0.54 | 0.89 | 0.54 | 0.54 | **0.08*** | 0.69 |  |  |  |  |  |  |  |  |  |  |
| **18h** | 0.75 | 0.75 | 0.74 | 0.52 | 0.83 | 0.55 | 0.41 | 0.71 | 0.13 | 0.8 |  |  |  |  |  |  |  |  |  |
| **19h** | 0.82 | 0.82 | 0.82 | 0.57 | 0.89 | 0.65 | 0.49 | 0.79 | 0.2 | 0.87 | **0*** |  |  |  |  |  |  |  |  |
| **21h** | 0.81 | 0.81 | 0.82 | 0.65 | 0.88 | 0.71 | 0.66 | 0.78 | 0.7 | 0.86 | 0.79 | 0.86 |  |  |  |  |  |  |  |
| **22h** | 0.81 | 0.8 | 0.82 | 0.66 | 0.87 | 0.72 | 0.67 | 0.78 | 0.7 | 0.85 | 0.78 | 0.85 | **0.02*** |  |  |  |  |  |  |
| **26h** | 0.81 | 0.8 | 0.81 | 0.66 | 0.86 | 0.72 | 0.68 | 0.77 | 0.71 | 0.83 | 0.78 | 0.84 | 0.1 | **0.01*** |  |  |  |  |  |
| **29h** | 0.9 | 0.9 | 0.9 | 0.77 | 0.93 | 0.82 | 0.74 | 0.87 | 0.78 | 0.93 | 0.89 | 0.93 | 0.89 | 0.89 | 0.88 |  |  |  |  |
| **32h** | 0.89 | 0.89 | 0.9 | 0.76 | 0.93 | 0.8 | 0.72 | 0.86 | 0.75 | 0.93 | 0.89 | 0.93 | 0.88 | 0.88 | 0.87 | **0*** |  |  |  |
| **33h** | 0.91 | 0.91 | 0.92 | 0.78 | 0.94 | 0.85 | 0.76 | 0.89 | 0.8 | 0.94 | 0.9 | 0.94 | 0.9 | 0.9 | 0.89 | **0*** | **0*** |  |  |
| **34h** | 0.89 | 0.89 | 0.89 | 0.8 | 0.9 | 0.85 | 0.82 | 0.87 | 0.84 | 0.89 | 0.88 | 0.9 | 0.88 | 0.88 | 0.87 | 0.9 | 0.89 | 0.9 |  |
| **35h** | 0.77 | 0.75 | 0.77 | 0.68 | 0.79 | 0.7 | 0.67 | 0.74 | 0.7 | 0.79 | 0.74 | 0.79 | 0.4 | 0.42 | 0.46 | 0.82 | 0.81 | 0.83 | 0.83 |

All sites are statistically differentiated (p<0.05) except for sites in bold text with asterisks. Significance tests are based on null distributions of haplotypes generated by 1023 permutations of haplotypes between each comparison of populations.

**Table S4** Pair-wise F_ST_ values between sites for the *Anopheles hinesorum* rpS9 locus.

|  | **10h** | **12h** | **13h** | **14h** | **18h** | **19h** | **20h** | **21h** | **22h** | **26h** | **32h** | **33h** | **34h** |
| --- | --- | --- | --- | --- | --- | --- | --- | --- | --- | --- | --- | --- | --- |
| **12h** | **0.04*** |  |  |  |  |  |  |  |  |  |  |  |  |
| **13h** | **0.02*** | 0.15 |  |  |  |  |  |  |  |  |  |  |  |
| **14h** | 0.12 | 0.17 | **0.02*** |  |  |  |  |  |  |  |  |  |  |
| **18h** | 0.46 | 0.47 | 0.61 | 0.73 |  |  |  |  |  |  |  |  |  |
| **19h** | 0.51 | 0.51 | 0.64 | 0.74 | **0*** |  |  |  |  |  |  |  |  |
| **20h** | 0.48 | 0.49 | 0.61 | 0.72 | **0.1*** | **0.03*** |  |  |  |  |  |  |  |
| **21h** | 0.54 | 0.59 | 0.69 | 0.8 | 0.88 | 0.86 | 0.85 |  |  |  |  |  |  |
| **22h** | 0.61 | 0.64 | 0.75 | 0.84 | 0.92 | 0.9 | 0.89 | **0.01*** |  |  |  |  |  |
| **26h** | 0.62 | 0.66 | 0.76 | 0.86 | 0.94 | 0.92 | 0.9 | **0.03*** | **0*** |  |  |  |  |
| **32h** | 0.54 | 0.60 | 0.68 | 0.81 | 0.92 | 0.87 | 0.84 | 0.9 | 0.95 | 0.97 |  |  |  |
| **33h** | 0.53 | 0.59 | 0.66 | 0.77 | 0.8 | 0.79 | 0.76 | 0.83 | 0.89 | 0.91 | **0*** |  |  |
| **34h** | 0.7 | 0.71 | 0.8 | 0.88 | 0.97 | 0.95 | 0.93 | 0.91 | 0.95 | 0.98 | 1 | 0.93 |  |
| **35h** | 0.57 | 0.59 | 0.68 | 0.76 | 0.81 | 0.81 | 0.79 | 0.62 | 0.68 | 0.7 | 0.8 | 0.77 | 0.2 |

All sites are statistically differentiated (p<0.05) except for sites in bold text with asterisks. Significance tests are based on null distributions of haplotypes generated by 1023 permutations of haplotypes between each comparison of populations.
